# Plasmapheresis reverses all side-effects of a cisplatin overdose – a case report and treatment recommendation

**DOI:** 10.1186/1471-2407-6-1

**Published:** 2006-01-04

**Authors:** Guenter Hofmann, Thomas Bauernhofer, Peter Krippl, Doris Lang-Loidolt, Sabine Horn, Walter Goessler, Walter Schippinger, Ferdinand Ploner, Herbert Stoeger, Hellmut Samonigg

**Affiliations:** 1 Division of Oncology, Department of Internal Medicine, Medical University of Graz, Auenbruggerplatz 15, 8036 Graz, Austria; 2 Ear, Nose and Throat University Hospital, Medical University of Graz, Auenbruggerplatz 26-28, 8036 Graz, Austria; 3 Division of Nephrology, Department of Internal Medicine, Medical University of Graz, Auenbruggerplatz 15, 8036 Graz, Austria; 4 Institute of Chemistry, Analytical Chemistry, Karl-Franzens-University of Graz, Universitätsplatz 1, 8010 Graz, Austria

## Abstract

**Background:**

Cisplatin is widely used as an antineoplastic agent since it is effective against a broad spectrum of different tumours. Nevertheless, it has several potential side effects affecting different organ systems and an overdose may lead to life-threatening complications and even death.

**Case presentation:**

We report on a 46-year old woman with non-small cell lung cancer who accidentally received 225 mg/m^2 ^of cisplatin, which was threefold the dose as scheduled, within a 3-day period. Two days later, the patient presented with hearing loss, severe nausea and vomiting, acute renal failure as well as elevated liver enzymes. In addition, she developed a severe myelodepression. After plasmapheresis on two consecutive days and vigorous supportive treatment, the toxicity-related symptoms improved and the patient recovered without any sequelae.

**Conclusion:**

To date, no general accepted guidelines for the treatment of cisplatin overdoses are available. Along with the experience from other published cases, our report shows that plasmapheresis is capable of lowering cisplatin plasma and serum levels efficiently. Therefore, plasma exchange performed as soon as possible can ameliorate all side effects of a cisplatin overdose and be a potential tool for clinicians for treatment. However, additional intensive supportive treatment-modalities are necessary to control all occurring side effects.

## Background

Cisplatin is a widely used antineoplastic agent which is effective against different types of tumours. In addition to its high antitumour activity, cisplatin is a drug with potential side effects including nephro-, neuro-, myelo- and ototoxicity, as well as liver damage and severe emesis. These toxicities are dose-dependent and dose- and therapy-limiting. The maximum tolerated single dose of cisplatin is considered to be 100 to 120 mg/m^2 ^per cycle and should be administered with adequate pre- and posthydration [1,2]. However, accidental overdose of cisplatin may occur despite all precautions and to date, no general accepted guidelines for the treatment of such cases are available.

## Case presentation

**Figure 1:**
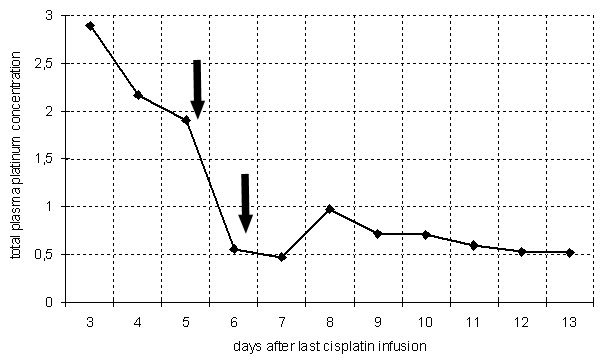
shows plasma platinum levels measured from day 3 to day 13 after the last cisplatin administration in mg platinum/kg plasma. Black arrows indicate the time of the two plasmaphereses, the black rhomboids the extent of platinum levels in mg/kg plasma.

**Figure 2:**
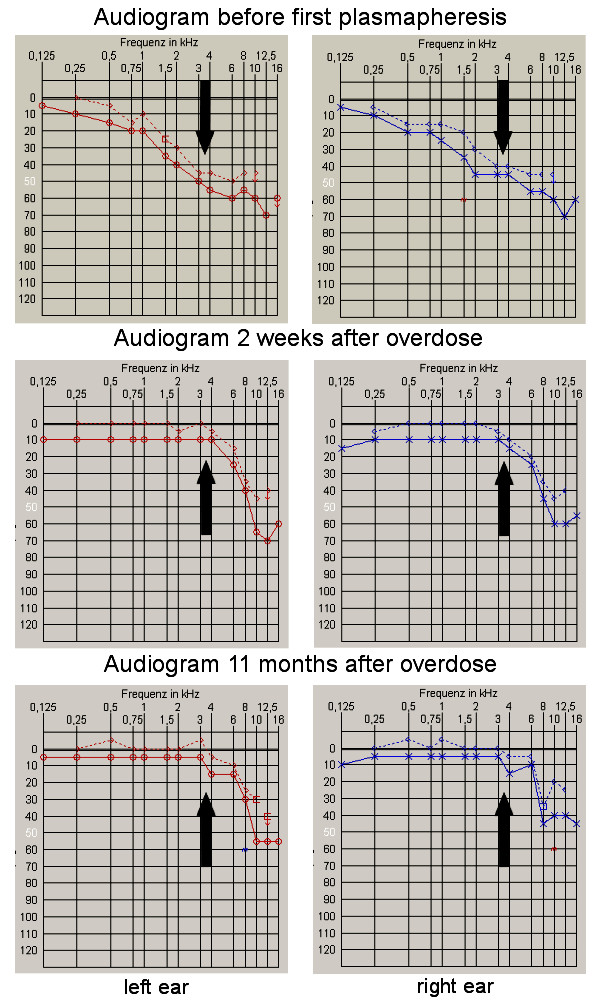
demonstrates the complete restitution of the hearing curves from 0.75 kilohertz to 6 kilohertz in the audiograms of the patient performed at three different times. On the x-axis the frequency in kilohertz is shown, the y-axis indicates the loudness in decibel. The arrows point at the changes in the curves.

We report on a 46-year-old female patient with histologically proven adenocarcinoma of the right lung. The patient underwent pneumonectomy and unilateral lymphadenectomy in December 2001. Because of histologically verified positive resection margins, it was decided to administer 4 cycles of a postoperative polychemotherapy regimen including cisplatin 25 mg/m^2^and etoposide 120 mg/m^2 ^on day 1, day 2 and day 3 followed by chest irradiation. Due to a prescription error, the patient received 75 mg/m^2 ^cisplatin per day, instead of 25 mg/m^2 ^resulting in a total dose of 225 mg/m^2 ^cisplatin within a 3-day period. The error was not noticed and the patient was discharged. Two days later, the patient presented with severe nausea and vomiting, loss of hearing, tinnitus and was unable to eat and drink. Due to the severity of these side effects, the medical records of the patient were reviewed and the cisplatin overdose was discovered. Supportive treatment including vigorous hydration, electrolyte replacement and antiemetic therapy was started immediately. Total platinum plasma levels which were determined daily showed highly elevated values (2.9 mg platinum per kg plasma). Therefore, plasmapheresis was performed on day 5 and 6 after the last administration of cisplatin to attempt to eliminate the drug. The platinum concentrations were measured by an Agilent 7500c inductively coupled plasma mass spectrometer (Agilent, Waldbronn, Germany) equipped with a PFA50 microconcentric nebulizer. The plasma samples were mineralisated microwave assisted with a mixture of nitric acid and hydrochloric acid (1 ml sample + 2 ml of nitric acid + 0.5 ml of hydrochloric acid + 1.5 ml ultrapure water) in an UltraCLAVE2 (EMLS, Leutkirch, Germany) at 250°C for 30 minutes before measurement. Plasmapheresis was carried out via a catheter placed in the right femoral vein and using a plasma filtration technique with Hemaplex BT 900/A Filters (Dideco, Mirandola, Italy). During each treatment session, 3 liters of plasma were removed and continuously substituted with 1.5 liter of solvent-detergent treated standardized pooled human plasma (Octaplas, Octapharma Vienna, Austria) and 1.5 liter of 5% human albumin (Immuno, Vienna, Austria). Anticoagulation therapy during plasmapheresis consisted of 3000 IU (international units) heparin administered as intravenous bolus and 1000 IU heparin per hour as continuous infusion. The plasma levels of total platinum showed a marked decline after plasma exchange at first, followed by a small rise and then a constant decline afterwards as demonstrated in Figure 1. After the first plasmapheresis, hearing loss and tinnitus improved gradually as well as renal failure. (The elevated laboratory findings are shown in Table1.) Severe myelosuppression required the administration of granulocyte-colony stimulating factor, two units of packed and irradiated erythrocytes as well as two units of packed, pooled and irradiated thrombocytes. In order to manage prolonged severe nausea and vomiting which lasted for 12 days, a complex antiemetic regimen including 5HT_3_-antagonists, dexamethasone, metoclopramide and neuroleptics was given. Finally, we were able to discharge the patient 18 days after onset of the overdose. She recovered completely from all toxicities including hearing loss which was proven clinically and by audiometry (Figure 2) except for a subclinical renal impairment remaining from the acute renal failure (Table2). Further antineoplastic therapy was continued with 2 cycles of a carboplatin-based regimen followed by chest irradiation. The patient showed no signs of relapse until March 2003, when a single brain metastasis was discovered and removed by surgery. In September 2003, recurrence of multiple brain metastases was detected and the patient died in January 2004 due to repeated progression of these metastases despite several treatment attempts. During the whole course of disease, no other metastatic sites and no late onset toxicities were observed in the patient.

## Discussion

In 1992, the first warning for all physicians and hospital pharmacists regarding erroneous cisplatin dosing was published [3] after the reporting of several cases of cisplatin overdose to a pharmaceutical company. Three possible mechanisms of errors were identified: total dose of one cycle divided over a period of days was given as a daily dose (as in our case), administration of cisplatin instead of carboplatin, and the prescription and administration of an overdose itself. Due to the fact that no antidote exists for cisplatin, a continuous effort has been made to protect patients from cisplatin induced toxicities since the early 1980's [1,2,4,5]. Besides prevention, a possible treatment option for a cisplatin overdose was identified as to try to directly eliminate cisplatin from body fluids.

**Figure 3:**
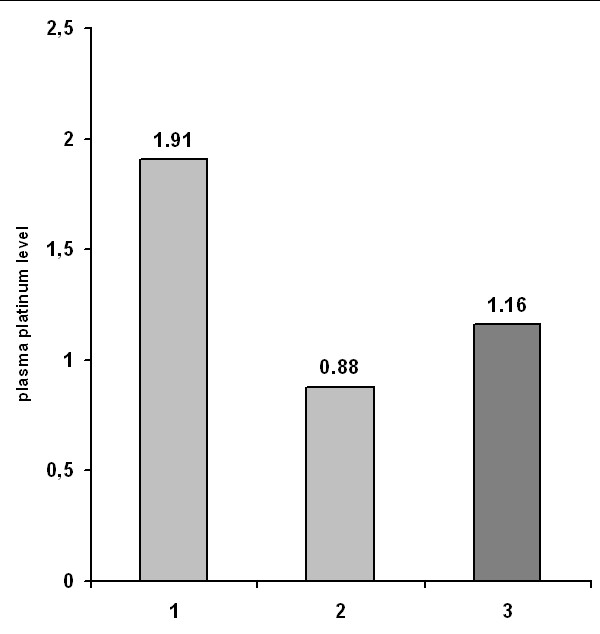
shows platinum levels in plasma samples taken one hour before and one hour after the first plasmapheresis and from the pooled plasma extracted by this plasmapheresis in mg/kg plasma. Numeration of colums: column 1: platinum level of plasma sample before plasmapheresis, column 2: platinum level of plasma sample after plasmapheresis, column 3: platinum level of extracted plasma.

Hemodialysis, for example, is able to reduce free cisplatin in plasma, but cisplatin binds to plasma proteins after administration very quickly and therefore cannot be further eliminated by this procedure [6,7]. In addition, it has been shown that there exists a rebound of cisplatin into plasma from an exchangeable pool shortly after hemodialysis. Plasmapheresis substantially removes plasma with its proteins and therefore the protein-bound fraction of cisplatin (see Figure 3), which seems to be a large part of this exchangeable pool of cisplatin distributed in the body. The same rebound-phenomenon as observed in hemodialysis exists after plasmapheresis (see Figure 1), but due to the better clearance of cisplatin, which is mostly plasma-protein bound in vivo, the platinum-pool is reduced more effectively.

The cisplatin-fraction which is distributed in this exchangeable pool, is mostly responsible for prolonged toxic effects [6,8]. In addition to our experience, data from the cases reported in literature (Table3) show that only plasmapheresis performed before day 12 after overdose leads to the complete recovery of the patients including hearing loss without sequelae. It seems that the delay in the start of treatment prolonges cisplatin distribution from this exchangeable pool into other body compartments and consequently causes irreversible damage [8]. Plasma exchange started even on day 12 after overdose was still able to reduce plasma platin levels on the one hand and improve clinical symptoms on the other hand [8]. Therefore, plasmapheresis should be performed in any case of a cisplatin overdose regardless of the time elapsed since overdose to at least attempt to improve the patient's condition of the patient. In two other published cases, plasmapheresis was performed together with administration of a chemoprotectant, but the role of the protectant in ameliorating symptoms in this cases remained unclear [9,10]. Chemoprotection itself is another approach of combatting therapy-induced side effects in general. Several cytoprotective agents have been tested with cisplatin-based therapies and have proven to reduce and even avoid toxic events. They all have the common feature of compounds containing sulfur. Their mechanism of action is thought to be free radical scavenging, covalent binding to the agent or both, but is still not entirely understood [1,2,4].

Usually, all these protectants should be given before or during the administration of cisplatin in order to be effective [11], but it was demonstrated that sodium thiosulfate (STS) administered 70 hours after an overdose had an effect in improving renal function [12]. Nevertheless, data exist that tumour cells are protected as well as normal tissue [2,4]. There is only little evidence that chemoprotectants reverse loss of hearing as well [2,4]. Actually, a recently published study showed that Amifostine is unable to protect patients from hearing loss if cisplatin is used at higher doses [13] and was unable to protect a child from severe toxicities [14]. Finally, a significant amount of cisplatin is eliminated in urine [8] and this pathway of elimination is dependent on renal output and hydration. Therefore, it is of utmost importance that a cisplatin overdose is discovered as soon as possible so that the appropriate treatment can be started without the least possible delay. The patient should be transferred to a specialised tumour center with a facility for plasma exchange. Furthermore, laboratory parameters including renal function, blood count, electrolytes, liver parameters and cisplatin levels should be determined on a daily basis. Hydration, electrolyte replacement, vigorous antiemetic treatment, audiometry at several timepoints and early administration of cytokines like G-CSF or GM-CSF are essential for treatment success as well. Despite all the treatment modalities discussed above the main goal has to be the prevention of such mistakes. Since the early 1990's more and more institutions, comprehensive cancer centers and cancer societies have been dealing with this matter and general recommendations and guidelines have been developed [5,15-17]. A possible solution could be the system we have been using on our ward since the overdose to prevent dosing errors. We established a control system for each level, starting with prescription, then preparation and administration of the drugs. A computer-based prescription system was implemented containing all dosing schedules to reduce dosing errors as well as mistakes in reading and writing. Additionally, every prescription for agents with possible severe side-effects such as cisplatin has to be double-checked by a second doctor. On the preparation level, the pharmacists in our central oncology pharmacy use a computer-software for their workbenches as well, which automatically check for errors and is again cross-checked by the pharmacists themselves. At last, the doctor who administers the infusion checks the identification of the patient, the prescription and preparation of the drug, affixes the drug label into the medical record and documents this with his signature. These steps were established according to published recommendations and guidelines [15-17] and summarised in standard operating procedures (SOP's) that are reevaluated at regular intervals. Since then, no further dosing error has happened in our ward. Nevertheless, even such complex procedures are not able to completely prevent such mistakes as published recently [18]. Therefore, physicians and pharmacists should be aware of this problem and strictly follow appropriate guidelines in order to give the best care possible to patients.

## Conclusion

Referring to our case and the cases reported [19,20], plasmapheresis performed as soon as possible should be considered as integral part of treatment when a cisplatin overdose occurs. Plasmapheresis itself has calculable risks [21-24] and is available in most hospitals with units for hemodialysis. The early administration of chemoprotectants, mainly sodium thiosulfate, should be taken in consideration as well [12], until the patient can be transferred to a specialised center with the possibility and experience of plasma exchange. However, the best strategy still remains to be the prevention of such errors since even these treatments may be unsuccessful [9,10]. We therefore recommend to evaluate, establish and implement the best possible and accurate control mechanisms on different levels for prescription, preparation and administration of chemotherapy agents in general and in cisplatin-based regimens in particular.

## List of abbreviations used

IU: international units

G-CSF: granulocyte-colony stimulating factor

GM-CSF: granulocyte-macrophage-colony stimulating factor

STS: sodium thiosulfate

SOP: standard operating procedure

## Competing interests

The author(s) declare that they have no competing interests.

## Authors' contributions

GH, TB, PK, WS, FP, HSt and HSa made important contributions to conception, data interpretation, drafting the manuscript and treatment of the patient. DLL performed the hearing tests including the audiograms, SH performed the plasmaphereses, WG did the platinum measurements. All authors read and approved the final manuscript.

## Pre-publication history

The pre-publication history for this paper can be accessed here:



## Figures and Tables

**Table 1:** laboratory findings

	admission day	worst findings	discharge day	normal range
GOT = AST (U/l)	29	29	4	< 19
GPT = ALT (U/l)	48	48	8	< 23
GGT = GGTP (U/l)	25	25	21	< 19
bilirubine (mg/dl)	0.6	0.6	0.4	0.1–1.2
creatinine (mg/dl)	2.6	3	1.2	0.6–1.3
BUN (mg/dl)	114	114	37	10–45
UA (mg/dl)	11.1	11.1	4.9	2.4–5.7
leuco (G/l)	10.62	0.47	25.42	4.4–11.3
Hb (g/dl)	12.9	8.9	10.3	12–15.3
plt (G/l)	218	14	45	140–440

GOT = AST: aspartate aminotransferase, GPT = ALT: alanine aminotransferase, GGT = GGTP: gamma glutamyl transferase, BUN: blood urea nitrogen, UA: uric acid, leuco: leucocyte count, Hb: hemoglobine, plt: platelet count

**Table 2:** laboratory findings demonstrating renal function

						normal range
day before/after overdose	-4	+5	+15	+50	+271	
GFR (ml/min/1.73 m^2^)	85	18	73	69	73	95–160
creatinine (mg/dl)	0.8	3	1.2	1.3	1.2	0.6–1.3
BUN (mg/dl)	32	114	37	40	47	10–45

GFR: glomerular filtration rate (on day +5, +15 and +271 GFR was estimated by using the Mayo-clinic formula [25]); BUN: blood urea nitrogen; day 0 indicates the last infusion day of the overdose

**Table 3:** treatment, delay to start of treatment and outcome of published cases

age	sex	dosis	delay	treatment	outcome	author
41	f	200 mg/m^2^	4 hours	HD	alive, n.s.	Brivet et al.
36	m	480 mg/m^2^	6 days	HD	alive, CRF, hearing loss	Schiller et al.
62	m	240 mg/m^2^	3 days	STS, HD	alive, n.a.	Delanian et al.
68	f	280 mg/m^2^	12 days	PPH, HD	alive, CRF, hearing loss	Chu et al.
54	f	205 mg/m^2^	6 days	HD	alive, n.a.	Lagrange et al.
59	m	300 mg/m^2^	3 days	PPH	alive, n.s.	Jung et al.
38	f	total 640 mg	4 days	NAC	dead	Sheik-Hamad et al.
14	f	360 mg/m^2^	70 hours	STS	alive, hearing loss	Erdlenbruch et al.
48	m	400 mg/m^2^	no delay	PPH, HD	alive, n.s.	Choi et al.
63	m	total 750 mg	no delay	HD, NAC, PPH	dead	Charlier et al.

HD: hemodialysis; STS: sodium thiosulfate; PPH: plasmapheresis; NAC: N-acetylcysteine; w.s.: with sequelae; n.s.: no sequelae; n.a.: not applicable; CRF: chronic renal failure
